# Supplementary material for: Population Genomics for Coral Reef Restoration—A Case Study of Staghorn Corals in Micronesia
Source: Evol Appl. 2025 Jun 23;18(6):e70115. doi: 10.1111/eva.70115 (PMC12185382; doi:10.1111/eva.70115)
Supplement: Supplementary file 1 — Data S1. [file EVA-18-e70115-s001.zip › eva70115-sup-0002-FigureS2.pdf]

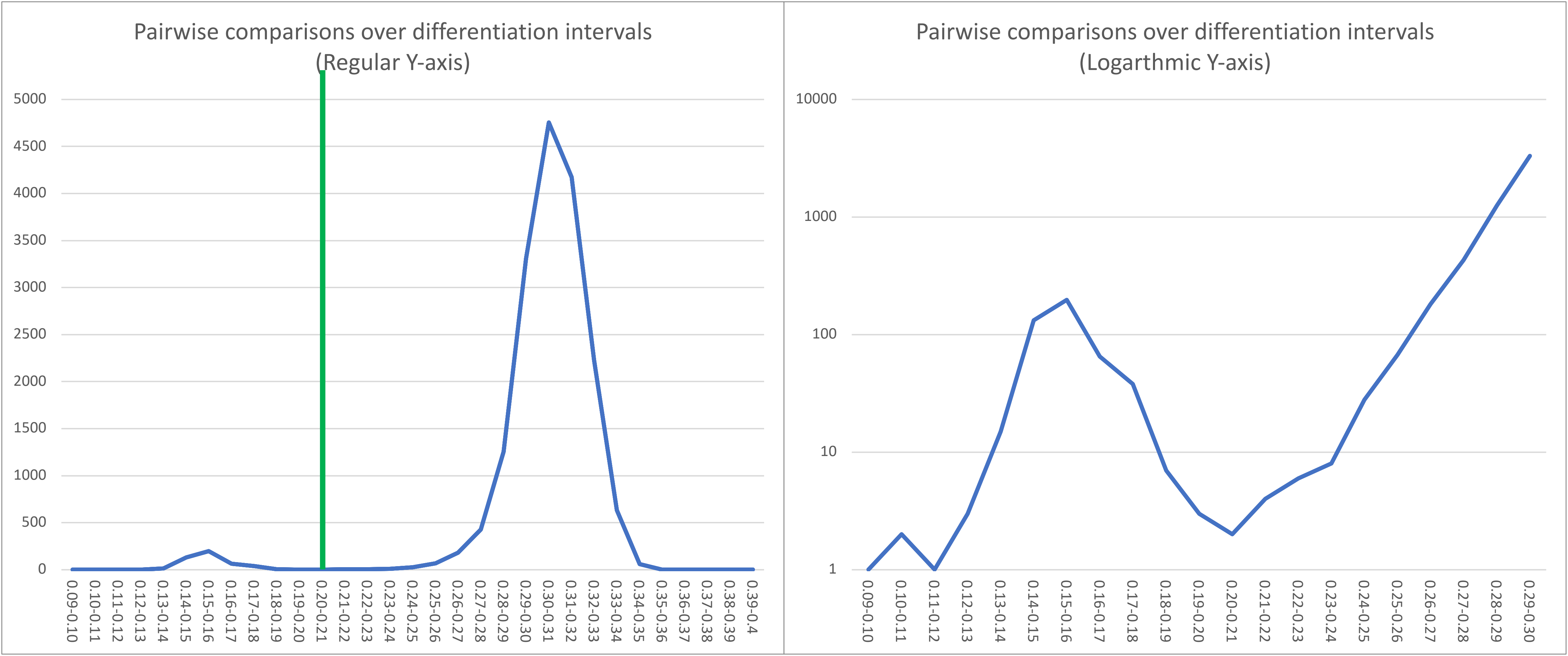
Pairwise comparisons over differentiation intervals
(Regular Y-axis)
5000
4500
4000
3500
3000
2500
2000
1500
1000
500
0
0.09-0.10
0.10-0.11
0.11-0.12
0.12-0.13
0.13-0.14
0.14-0.15
0.15-0.16
0.16-0.17
0.17-0.18
0.18-0.19
0.19-0.20
0.20-0.21
0.21-0.22
0.22-0.23
0.23-0.24
0.24-0.25
0.25-0.26
0.26-0.27
0.27-0.28
0.28-0.29
0.29-0.30
0.30-0.31
0.31-0.32
0.32-0.33
0.33-0.34
0.34-0.35
0.35-0.36
0.36-0.37
0.37-0.38
0.38-0.39
0.39-0.4
Pairwise comparisons over differentiation intervals
(Logarthmic Y-axis)
10000
1000
100
10
1
0.09-0.10
0.10-0.11
0.11-0.12
0.12-0.13
0.13-0.14
0.14-0.15
0.15-0.16
0.16-0.17
0.17-0.18
0.18-0.19
0.19-0.20
0.20-0.21
0.21-0.22
0.22-0.23
0.23-0.24
0.24-0.25
0.25-0.26
0.26-0.27
0.27-0.28
0.28-0.29
0.29-0.30
