# Supplementary material for: Population Genomics for Coral Reef Restoration—A Case Study of Staghorn Corals in Micronesia
Source: Evol Appl. 2025 Jun 23;18(6):e70115. doi: 10.1111/eva.70115 (PMC12185382; doi:10.1111/eva.70115)
Supplement: Supplementary file 1 — Data S1. [file EVA-18-e70115-s001.zip › eva70115-sup-0006-AppendixS1.docx]

# Figures & Tables

All figures were submitted as individual pdf files and figure legends included in this Figures-Tables file. Main tables were included in the main text, supplementary tables were included in the Figures-Tables file below. If a main figure contained a table, the table was left in the main text while the figure was submitted separately.

## Figures

### Main:

FIGURE 1: Location of the five sampling sites of *A.* cf. *pulchra* on Guam and Saipan

FIGURE 2: F_ST_ differentiation and Isolation by distance.

FIGURE 3: Principal coordinate analysis (PCoA)

FIGURE 4: Relatedness within and among populations

FIGURE 5: Migration among Guam populations

FIGURE 6: Symbionts ddRAD

#### Supplementary:

Fig. S1: Phylogenomics

Fig. S2: Clonality

Fig. S3: Spatial Genetic Structure

Fig. S4: Admixture

Fig. S5: Symbiont ddRAD vs. ITS2 metabarcoding

Fig. S6: Extent of limestone terrain on Guam

## Tables

### Main:

TABLE 1: Clonality and Genetic Diversity statistics

TABLE 2: Number of outlier loci found between populations as detected by BayPAss

#### Supplementary:

Table S1: GPS location of populations and number of *Acropora* samples

Table S2: SNP-based Diversity statistics

Table S3: F_ST_, G_ST_ and D_EST_ pairwise population differentiation

Table S4: IbD for different measures of pairwise differentiation

Table S5: AMOVAs

## Figures

### Main:

FIGURE 1: Location of the five sampling sites of *A.* cf. *pulchra* on Guam and Saipan.

**Submitted as Figure1_231211.pdf**

The large island right below Saipan is Tinian, tiny Aguijan is right below, and Rota is right in between Aguijan and Guam. Populations are color-coded as follows: Dåno’ = orange, Hågat = red, Aniguak = light blue, Urunao = dark blue; Saipan = green; Tokcha’ = yellow.

FIGURE 2: F_ST_ differentiation and Isolation by distance.

**Fig.2a) Submitted as Figure2_v2.pdf**

a) Isolation-by-distance analyses based on F_ST_ /(1-F_ST_) over log transformed oceanographic distances for all populations but Tokcha’. A strong and significant IbD pattern was observed across all populations (r^2^ = 0.587; p < 0.05; all data points above and the solid trendline). The IbD pattern detected among Guam populations (white diamonds and the dotted trendline) was not significant (r^2^ = 0.768; p = 0.161).

**Fig.2b) is a table in the main text.**

b) Pairwise F_ST_ values (below triangle) and associated p-values (above triangle) between populations (as calculated by GenoDive).

All comparisons in bold on white ground have a significant p value after sequential Bonferroni correction (p = 0.05). Tokcha’ was excluded from these analyses due to its low number of unique genotypes (n=2).

FIGURE 3: Principal coordinate analysis (PCoA)

**Fig.3a) Submitted as Figure3.231209.pdf**

This PCoA is based on covariance matrices generated by the ANGSD subprogram ngsAdmix. Populations are color-coded as follows: Dåno’ = orange, Hågat = red, Aniguak = light blue, Urunao = dark blue; Saipan = green; Tokcha’ = yellow.

FIGURE 4: Relatedness within and among populations

**Fig.4) Submitted as Figure4.240916.pdf**

Relatedness (1-3^rd^ degree) among samples within and across populations as outlined in method section 2.4. Percentages of closely related individuals (rab > 0.09375, i.e. third-degree relatives and closer) are outlined below the diagonal.

FIGURE 5: Migration among Guam populations

**Fig.5a) Submitted as Figure4.MigMap_Apul2.pdf**

Arrow color and width indicate the proportion of individuals in each population that originated in the population itself and in other populations, as calculated with BA3-SNPs. Specific values and confidence intervals are given in the table below.

**Fig.5b) is a table in the main text**

Proportion of individuals in each population that originated in the population itself and in other populations, as calculated with BA3-SNPs. Rows: Assessed population; Columns: Population of origin. Values in brackets indicate 95% confidence interval.

FIGURE 6: Dominant Photo-symbionts

**Fig.6a) Submitted as Figure5_reordered.pdf**

Bar plot representing the relative proportions of ddRAD reads producing highly unique matches to transcriptomes of four different genera of algal symbionts, Symbiodinium, Breviolum, *Cladocopium*, and *Durusdinium* (formerly Clades A-D, respectively).

**Fig.6b) is a table in the main text**

b) The distribution of colonies dominated by either *Cladocopium* or *Durusdinium* was significantly uneven among populations (p < 0.0001).

###### Supplementary:

Fig. S1: Phylogenomics

**Submitted as FigS1.RAxML_bipartitions.result.tre.pdf**

Phylogenetic tree used to identify *Acropora cf. pulchra* specimen*.* Green = *A. cf. pulchra*, black = other *Acropora* species.

FIGURE S2: Clonality

**a) Submitted as FigS2a.231211.4.pdf**

Hierarchical cluster dendrogram based on pairwise identity-by-state (IBS) values from ANGSD for 188 samples, including 18 technical replicates, indicated by gray boxes: 9 samples were sequenced twice and included here separately (as technical replicates) and combined (as in subsequent analyses). Technical replicates and gap analysis (below) were used to determine a threshold (indicated by the dashed red line) to distinguish clones (below threshold) from unique genotypes (above threshold).

**b) Submitted as FigS2b.pdf**

Clonality threshold justification. The distribution of pairwise comparisons is shown over differentiation intervals in 0.01 increments. The version on the left shows an overview with a regular Y-axis while the graph on the right has a logarithmic Y axis to display the gap between clonal comparisons on the left and differentiation due to sexual reproduction on the right.

FIGURE S3: Spatial Genetic Structure

**Submitted as FigS3.pdf**

Average pairwise kinship (Loiselle et al 1995) per distance interval, i.e. every 10m, for the four main Guam populations in the complete dataset including clones (n = 129) and the population genetics dataset, excluding clones (n = 55).

Fig. S4: Admixture

**Submitted as FigS4.240912.pdf**

The proportion of admixture in each individual sample from K=2 (A), on the left) and K=3 (B), on the right) genetic clusters is shown. Both admixture plots emphasize the difference between Guam and Saipan. In addition, both plots indicate more admixture from Saipan in Northern Guam populations (Urunao & Aniguak) compared to Southern Guam populations (Dåno’ and Hågat).

FIGURE S5: Symbiont ddRAD vs. ITS2 metabarcoding

**Submitted as FigS5.pdf**

Bar plot representing the relative proportions of ITS reads aligning to four different genera of algal symbionts, *Symbiodinium*, *Breviolum*, *Cladocopium*, and *Durusdinium* (formerly Clades A-D, respectively).

Among these 20 samples, 7 samples had virtually identical results with both methods (35%), 7 ddRAD libraries failed to detect minor genera identified with ITS2 (4x C, 2x D & 1x A) and 6 ITS2 libraries failed to identify minor genera identified with ddRAD (3x C & 3x D).

In addition, ITS2, suggested the presence of trace proportions (<0.02%) of symbiont clades E, F, G, H and I.

FIGURE S6: Extent of limestone terrain on Guam

**Submitted as FigS6.Pago-Adelup Fault.pdf**

Figure 1 from Taborosi et al (2004): 1. Location of Guam and the extent of its limestone terrain over the two provinces separated by the Pago-Adelup Fault.

## Tables

### Main (in the main text):

TABLE 1: Clonality and Genetic Diversity statistics.

TABLE 2: Number of putative loci under selection between populations

###### Supplementary:

Table S1: Sampling locations and samples

| Sampling site | Latitude | Longitude | *Acropora* samples  sequenced | Identified as  *A.* cf. *pulchra* | Included in  analyses |
| --- | --- | --- | --- | --- | --- |
| Dåno’ | 13.24589 | 144.68489 | 61 | 50 | 26 |
| Hågat | 13.38322 | 144.65169 | 49 | 42 | 30 |
| Aniguak | 13.47970 | 144.74590 | 50 | 50 | 32 |
| Urunao | 13.63672 | 144.84527 | 45 | 45 | 41 |
| Tokcha’ | 13.36865 | 144.77541 | 21 | 21 | 21 |
| Saipan | 15.20568 | 145.74078 | 41 | 25 | 20 |
| **Total** |  |  | **267** | **233** | ***170** |

GPS locations of populations and number of *Acropora* samples collected from six sites on Guam and Saipan (n = 267) and number of *A.* cf. *pulchra* samples included in the present study.  *Excluding 9 x2 (18) technical replicates

Table S2: SNP-based diversity statistics.

|  | | | **Genetic Diversity (SNPs)** | | | | | |
| --- | --- | --- | --- | --- | --- | --- | --- | --- |
| **Population** | **N** | **N_G_** | **N_A-SNPs_** | **N_A-SNPs_ -eff** | **π** | **H_O_** | **H_E_** | **F_IS_** |
| Dåno’ | 26 | 7 | 1.46 | 1.264 | 0.131 | **0.113** | 0.113 | **0.038** |
| Hågat | 30 | 10 | 1.59 | 1.281 | 0.134 | 0.110 | **0.123** | **0.062** |
| Aniguak | 32 | 14 | 1.60 | 1.267 | 0.128 | 0.107 | **0.120** | **0.060** |
| Urunao | 41 | 24 | 1.66 | 1.275 | 0.121 | 0.102 | 0.117 | **0.077** |
| *Tokcha’** | *21* | ***2*** | 1.33 | 1.236 | *0.120* | *0.105* | *0.083* | ***0.023*** |
| Saipan* | 20 | *17* | 1.67 | 1.289 | *0.137* | *0.112* | *0.130* | ***0.084*** |
| **Overall** | 170 | 74 | 2.00 | 1.238 | 0.132 | 0.108 | 0.131 | ****0.144** |

SNP-based diversity statistics, calculated with GenoDive for the four main Guam populations (Dåno’, Hågat, Aniguak and Urunao), Tokcha’, Saipan and overall.

N = Number of samples, N_G_ = Number of unique genotypes; N_A-SNPs_ = Number of Alleles over SNPs; N_A-SNPs_-eff = effective Number of Alleles over SNPs; π = Pi = Nucleotide Diversity.

H_o_ = Observed heterozygosity; H_e_ = Expected heterozygosity; F_is_ = Inbreeding coefficient; All F_IS_ values are significant (p < 0.05).

*Tokcha’ and Saipan were sampled differently, which limits their comparability (italic font).

**F_IS_ overall is affected by population structure, the F_IS_ value across the 4 main Guam populations (0.059) therefore represents a better estimate of *A.* cf. *pulchra* inbreeding on Guam.

Table S3: Pairwise differentiation among populations, calculated as F_ST_, G’_ST_ and D_EST_.

|  | Southern Guam | | Northern Guam | |  |
| --- | --- | --- | --- | --- | --- |
| 1. **F_ST_** | Dåno’ | Hågat | Aniguak | Urunao | Saipan |
| Dåno’ |  | 0.333 | ***0.006*** | **0.003** | **0.003** |
| Hågat | 0.005 |  | **0.002** | ***0.008*** | **0.001** |
| Aniguak | ***0.027*** | **0.016** |  | 0.120 | **0.001** |
| Urunao | **0.028** | ***0.014*** | 0.005 |  | **0.001** |
| Saipan | **0.026** | **0.028** | **0.029** | **0.021** |  |

| 1. **G’_ST_** | Dåno’ | Hågat | Aniguak | Urunao | Saipan |
| --- | --- | --- | --- | --- | --- |
| Dåno’ |  | 0.183 | **0.005** | **0.001** | **0.001** |
| Hågat | 0.012 |  | **0.008** | **0.015** | **0.001** |
| Aniguak | **0.032** | **0.016** |  | 0.133 | **0.001** |
| Urunao | **0.034** | **0.012** | 0.004 |  | **0.001** |
| Saipan | **0.037** | **0.028** | **0.030** | **0.020** |  |

| 1. **D_EST_** | Dåno’ | Hågat | Aniguak | Urunao | Saipan |
| --- | --- | --- | --- | --- | --- |
| Dåno’ |  | 0.184 | **0.001** | **0.002** | **0.001** |
| Hågat | 0.003 |  | **0.004** | **0.013** | **0.001** |
| Aniguak | **0.007** | **0.004** |  | 0.135 | **0.001** |
| Urunao | **0.008** | **0.003** | 0.001 |  | **0.001** |
| Saipan | **0.009** | **0.007** | **0.007** | **0.005** |  |

Pairwise differentiation among populations, calculated with GenoDive (lower triangle) and corresponding p-values (upper triangle). Significant p-values/differentiations are indicated in bold. All pairwise comparisons were significant with all three measures of differentiation, but Dåno’ vs. Hågat and Aniguak vs. Urunao, which were never significant. These comparisons constitute within-metapopulation comparisons on Guam, i.e. among Southern and Northern populations, as indicated above Table S4 A).

Pairwise differentiation between islands: F_ST_= 0.024; G’_ST_ = 0.022; D_est_ = 0.005; p < 0.001 for all three.

Table S4: IbD distance results for different measures of pairwise differentiation (Table S3) and regular oceanographic distance as well as log-transformed distances.

| **Measure** | **Log Distance** | | **Oceanographic Distance** | |
| --- | --- | --- | --- | --- |
|  | **r2** | **p** | **r2** | **p** |
| **F_ST_/(1-F_ST_)** | 0.587 | 0.021 | 0.414 | 0.009 |
| **F_ST_** | 0.588 | 0.025 | 0.414 | 0.008 |
| **G’_ST_** | 0.455 | 0.029 | 0.384 | 0.008 |
| **D_EST_** | 0.482 | 0.024 | 0.384 | 0.009 |

Table S5: AMOVAs

A) Overall

| **Source of Variation** | **%var** | **F-value** | **P-value** |
| --- | --- | --- | --- |
| Within Individual | 93.8 | 0.062 | – |
| Among Individual | 3.3 | 0.034 | **<0.001** |
| **Among Population** | **1.4** | **0.014** | **<0.001** |
| **Among Islands** | **1.5** | **0.015** | **0.017** |
| Within Individual | 93.6 | 0.064 | – |
| Among Individual | 4.1 | 0.042 | **<0.001** |
| **Among Islands** | **2.4** | **0.024** | **<0.001** |
| Within Individual | 94.6 | 0.054 | **—** |
| Among Individual | 3.3 | 0.034 | **<0.001** |
| **Among Populations** | **2.1** | **0.021** | **<0.001** |

B) Guam main 4 populations only:

| **Source of Variation** | **%var** | **F-value** | **P-value** |
| --- | --- | --- | --- |
| Within Individual | 96.0 | 0.040 | – |
| Among Individual | 2.0 | 0.021 | <0.001 |
| **Among Populations** | **0.7** | **0.007** | **0.029** |
| **North-South** | **1.2** | **0.012** | **<0.001** |
| Within Individual | 96.0 | 0.040 | – |
| Among Individual | 2.4 | 0.024 | <0.001 |
| **North-South** | **1.6** | **0.016** | **<0.001** |
| Within Individual | 96.5 | 0.035 | – |
| Among Individual | 2.0 | 0.021 | <0.001 |
| **Among Populations** | **1.5** | **0.015** | **<0.001** |
