# Supplementary figures and images for: Population Genomics for Coral Reef Restoration—A Case Study of Staghorn Corals in Micronesia
Source: Evol Appl. 2025 Jun 23;18(6):e70115. doi: 10.1111/eva.70115 (PMC12185382; doi:10.1111/eva.70115)

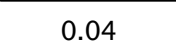

Supplement: Supplementary file 1 — Data S1. [file EVA-18-e70115-s001.zip › eva70115-sup-0001-FigureS1.pdf]

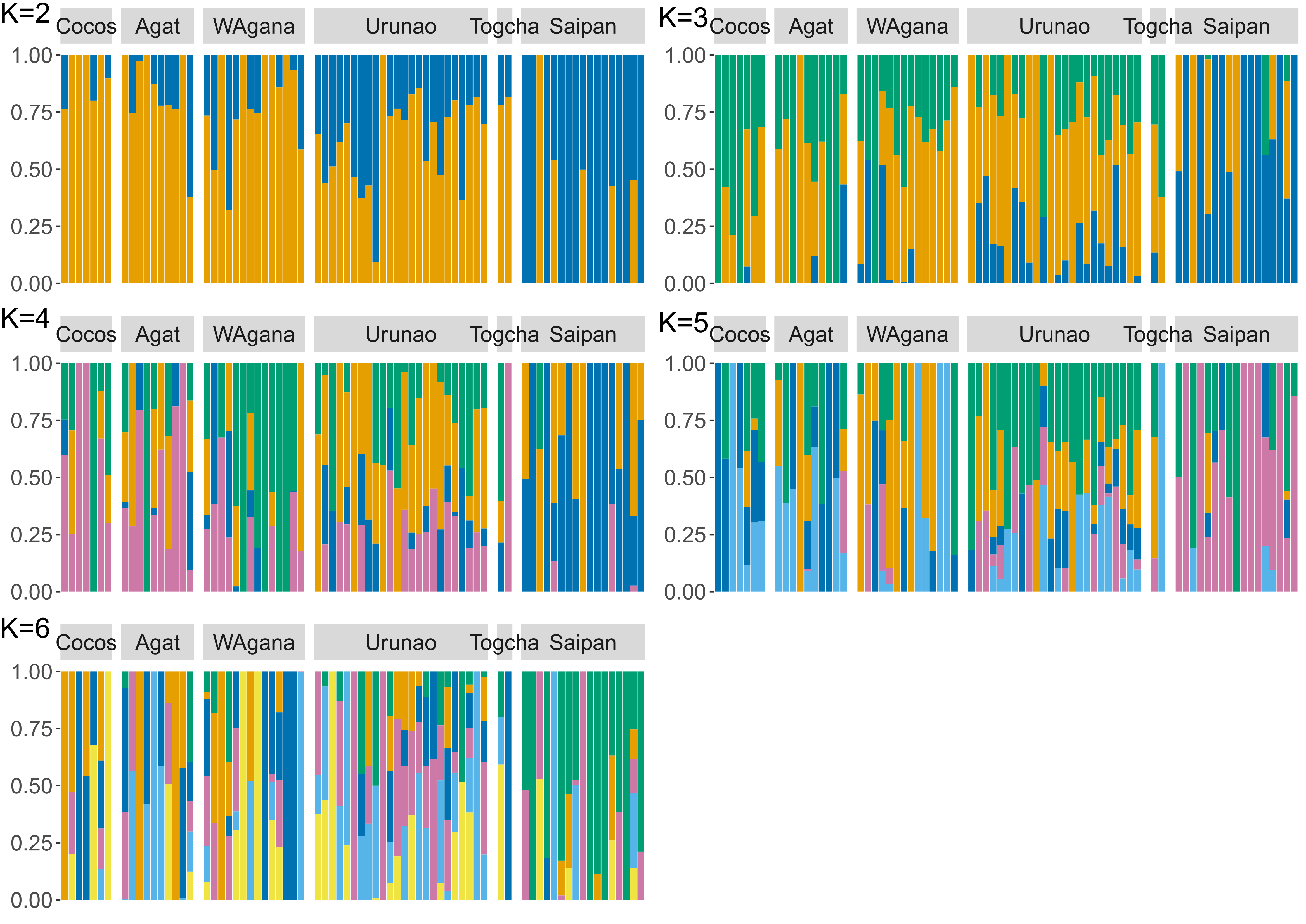

Supplement: Supplementary file 1 — Data S1. [file EVA-18-e70115-s001.zip › eva70115-sup-0003-FigureS3.png]

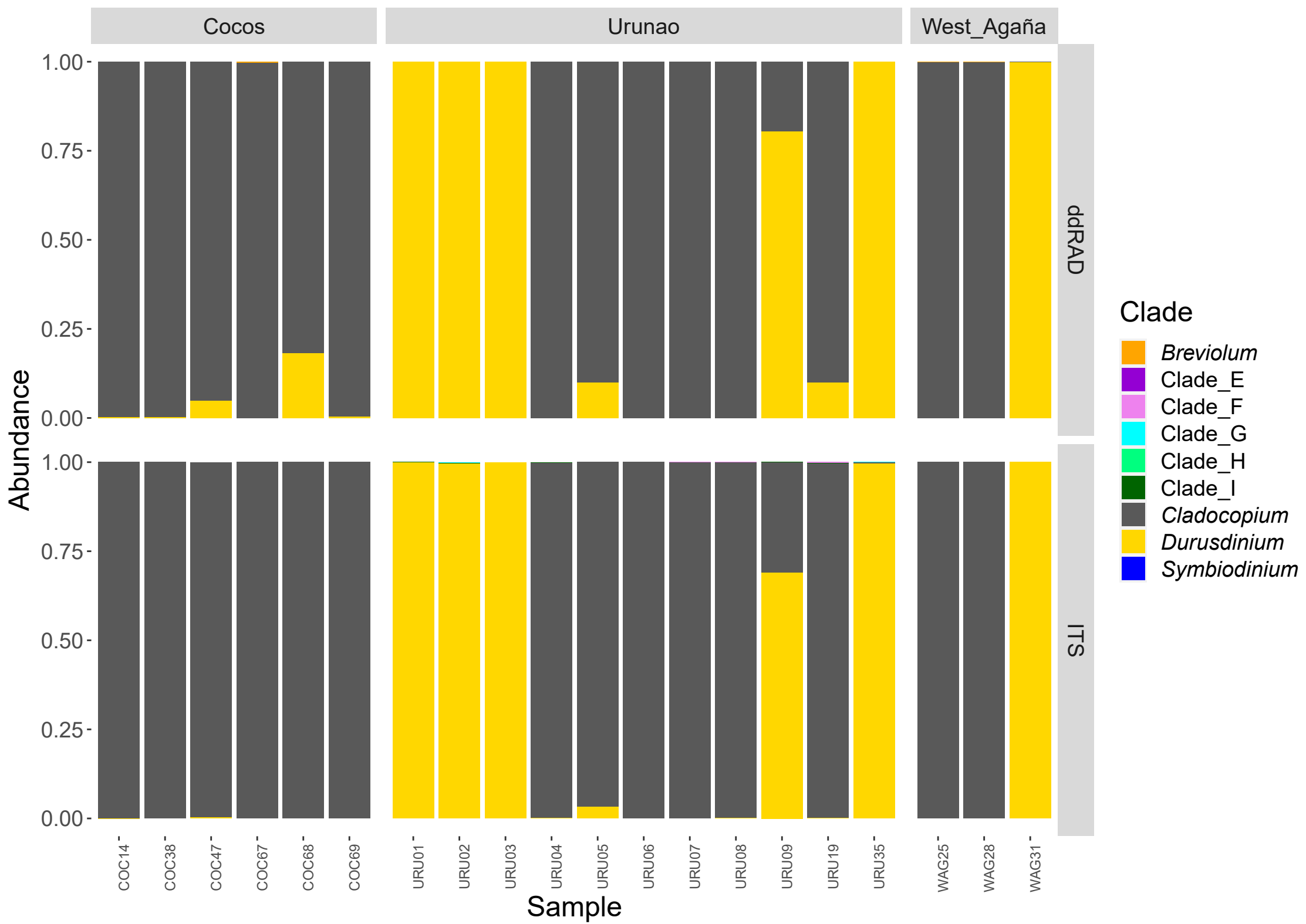

Supplement: Supplementary file 1 — Data S1. [file EVA-18-e70115-s001.zip › eva70115-sup-0004-FigureS4.pdf]
